# Microwave-Assisted Deep Eutectic Solvent Extraction, Structural Characteristics, and Biological Functions of Polysaccharides from Sweet Tea (*Lithocarpus litseifolius*) Leaves

**DOI:** 10.3390/antiox11081578

**Published:** 2022-08-15

**Authors:** Ding-Tao Wu, Meng-Xi Fu, Huan Guo, Yi-Chen Hu, Xiao-Qin Zheng, Ren-You Gan, Liang Zou

**Affiliations:** 1 Key Laboratory of Coarse Cereal Processing (Ministry of Agriculture and Rural Affairs), Sichuan Engineering & Technology Research Center of Coarse Cereal Industralization, School of Food and Biological Engineering, Chengdu University, Chengdu 610106, China; 2 Institute of Food Processing and Safety, College of Food Science, Sichuan Agricultural University, Ya’an 625014, China; 3 Research Center for Plants and Human Health, Institute of Urban Agriculture, Chinese Academy of Agricultural Sciences, Chengdu 610213, China

**Keywords:** sweet tea, polysaccharide, microwave-assisted deep eutectic solvent extraction, physicochemical characteristics, biological activity

## Abstract

The leaf of sweet tea (*Lithocarpus litseifolius*) is widely used as an edible and medicinal plant in China, which is rich in bioactive polysaccharides. In order to explore and promote the application of sweet tea polysaccharides in the functional food industry, the microwave-assisted deep eutectic solvent extraction (MDAE) of polysaccharides from sweet tea leaves was optimized, and the structural properties and biological functions of sweet tea polysaccharides prepared by MDAE (P-DM) were investigated and compared with that of hot water extraction (P-W). The maximum yield (4.16% ± 0.09%, *w/w*) of P-DM was obtained under the optimal extraction conditions (extraction time of 11.0 min, extraction power of 576.0 W, water content in deep eutectic solvent of 21.0%, and liquid–solid ratio of 29.0 mL/g). Additionally, P-DM and P-W possessed similar constituent monosaccharides and glycosidic bonds, and the homogalacturonan (HG) and arabinogalactan (AG) might exist in both P-DM and P-W. Notably, the lower molecular weight, higher content of total uronic acids, and higher content of conjugated polyphenols were observed in P-DW compared to P-W, which might contribute to its much stronger in vitro antioxidant, anti-diabetic, antiglycation, and prebiotic effects. Besides, both P-DW and P-W exhibited remarkable in vitro immunostimulatory effects. The findings from the present study indicate that the MDAE has good potential to be used for efficient extraction of bioactive polysaccharides from sweet tea leaves and P-DM can be developed as functional food ingredients in the food industry.

## 1. Introduction

*Lithocarpus litseifolius* (Hance) Chun, also named sweet tea in Chinese folk, is widely used as an edible and medicinal plant [1]. The leaf of sweet tea has been approved as a new food material by the National Health and Family Planning Commission of China, which has also been utilized as an herbal tea to prevent and manage diabetes in the folk for a long history [1,2]. Generally, the leaf of sweet tea is rich in active ingredients, such as polysaccharides, flavonoids, phenolic acids, and triterpenoids [1,3,4,5,6,7,8]. Therefore, owing to these bioactive ingredients, it possesses multiple health benefits, such as antioxidant, anti-inflammatory, anti-diabetic, anti-cancer, anti-hypertensive, anti-microbial, cardioprotective, and hepatoprotective effects [1]. A large number of studies have shown that natural polysaccharides from edible and medicinal plants possess a variety of biological activities [9,10,11,12], which have attracted increasing attention in the functional food industry. However, the knowledge about the chemical structures and biological functions of sweet tea polysaccharides is still limited [5], which requires to be deeply investigated so as to promote its applications in the functional food industry.

Generally, the extraction procedure is regarded as one of the most important processing approaches for the research and development as well as the production of functional foods derived from natural polysaccharides. Because the extraction yield, chemical properties, and functional/bioactive properties of polysaccharides extracted from edible and medicinal plants are always varied by different extraction procedures [13,14,15,16]. Therefore, the extraction optimization of bioactive polysaccharides from sweet tea leaves is necessary for the deep processing and application of sweet tea polysaccharides in the functional food industry. Water is commonly utilized as the extraction solvent for the preparation of natural polysaccharides. Although water is easily available, it always has a low extraction efficiency [17]. Besides, the alkaline or acidic buffer has been utilized for the enhancement of extraction efficiency. However, these buffers are always unfriendly to the environment [17]. More recently, the deep eutectic solvent (DES), consisting of a hydrogen bonding acceptor and a hydrogen bonding donor, has attracted increasing attention to be applied as a new and green solvent for extracting natural polysaccharides due to its high extraction efficiency [17,18,19,20,21,22]. Besides, compared with other extraction solvents, DES possesses some merits of low cost, little or no toxicity, biodegradability, and recyclability [23,24]. Notably, natural polysaccharides extracted by DES always possess higher extraction yield and higher bioactivity than those of traditional extraction solvents [20,21,25]. Furthermore, compared with traditional hot water extraction, microwave-assisted extraction also has several advantages of short extraction time, high extraction efficiency, low solvent consumption, and low energy consumption [26]. Therefore, the combined use of microwave-assisted extraction and DES-assisted extraction could be a highly efficient and eco-friendly procedure for the preparation of natural polysaccharides.

A recent study has indicated that polysaccharides extracted from sweet tea leaves by microwave-assisted deep eutectic solvent extraction (MDAE) possess favorable biological activities [5]. Therefore, in order to further promote the potential applicability of MDAE for extracting bioactive polysaccharides from sweet tea leaves, the extraction conditions of MDAE were carefully optimized by using Box-Behnken Design in the present study. Subsequently, the structural properties and biological functions of sweet tea polysaccharides prepared by MDAE (P-DM) were investigated and compared with that of hot water extraction (P-W).

## 2. Materials and Methods

### 2.1. Materials and Chemicals

Dried leaves of *L. litseifolius* were purchased from Sichuan Mu Jiang Ye Ke Tea Co., Ltd. (Chengdu, China). The fresh leaves of *L. litseifolius* were dried with an air-dryer (DHG-9246A, Jinghong Experimental Equipment Co., Ltd., Shanghai, China) at the temperature of 50 °C as described by the company. The sweet tea was put into a mill and pulverized into powder. Finally, the powder was screened by an 80-mesh sieve and stored at the temperature of −20 °C for further analysis.

Choline chloride and ethylene glycol were purchased from KESHI (Chengdu, China). 2,2-Diphenyl-1-picrylhydrazyl (DPPH), vitamin C (*Vc*), 3-ethylbenzthiazoline-6-sulphonic acid (ABTS), butylated hydroxytoluene (BHT), Griess reagent, lipopolysaccharide (LPS), 3-(4,5-dimethylthiazol-2-yl)-2,5-diphenyl tetrazolium bromide (MTT), aminoguanidine (AG), and α-glucosidase (10 U/mg) were purchased from Sigma-Aldrich (St. Louis, MO, USA). Thermo-stable α-amylase (40,000 U/g) was purchased from Solarbio (Beijing, China). ELISA kits for interleukin-6 (IL-6) and tumor necrosis factor-α (TNF-α) were obtained from NeoBioscience (Shenzhen, China).

### 2.2. Extraction and Isolation of Polysaccharides from Sweet Tea Leaves

#### 2.2.1. Pretreatment of Raw Materials

The powder of sweet tea leaves (50.0 g) was pre-treated with 80% (*v/v*) of ethanol (500.0 mL) to remove most of the alcohol-soluble constituents by an ultrasonic cleaner (SB-800DTD, Ningbo Scientz Biotechnology Co., Ltd., Ningbo, China). The ultrasound power, extraction time, and temperature were set as 640 W, 30 min, and 25 °C, respectively. Furthermore, the extracted residues were utilized for the preparation of polysaccharides.

#### 2.2.2. Hot Water Extraction

Hot water extraction (HWE) was conducted as previously reported [5]. 5.0 g of pre-treated sweet tea leaves were mixed with 100.0 mL of water at 95 °C for 2.0 h. After centrifugation (4500× *g*, 15 min), the supernatant was collected for the removal of soluble starch and soluble proteins by thermal-stable α-amylase (5 U/mL) and pancreatin (1 U/mL), respectively. Thereafter, polysaccharides in the supernatant were isolated by the combined use of graded alcohol precipitation (the final concentration of ethanol, 72%, *v/v*) and membrane separation (the molar mass cutoff, 30 kDa). Finally, polysaccharides extracted from the leaves of sweet tea by hot water extraction (P-W) were obtained by freeze-drying and stored at the temperature of 20 °C for further analysis.

#### 2.2.3. Microwave-Assisted Deep Eutectic Solvent Extraction

The DES extraction solvent was prepared as previously optimized, which was composed of choline chloride (ChCl) and ethylene glycol (EG) in a molar ratio of 1:2 [24]. The microwave-assisted deep eutectic solvent extraction (MDAE) was conducted by a laboratory-scale microwave device (MKX-H1C1A, Shandong, China).

Firstly, the single factor experimental design was carried out to evaluate the effects of extraction time (4, 6, 8, 10, 12 min), extraction power (320, 400, 480, 560, 640 W), content of water in DES (10, 20, 30, 40, 50%), and liquid–solid ratio (20, 30, 40, 50, 60, mL/g) on the extraction yield of polysaccharides from sweet tea leaves, respectively. Briefly, for the optimization of extraction time, the extraction power, content of water in DES, and liquid–solid ratio were set as 480 W, 30%, and 30 mL/g, respectively; for the optimization of extraction power, the extraction time, content of water in DES, and liquid–solid ratio were set as 10 min, 30%, and 30 mL/g, respectively; for the optimization of content of water in DES, the extraction time, the extraction power, and liquid–solid ratio were set as 10 min, 560 W, and 30 mL/g, respectively; for the optimization of liquid–solid ratio, the extraction time, the extraction power, and content of water in DES were set as 10 min, 560 W, and 20%, respectively. After the extraction, the supernatant was applied to de-starch and de-proteins as mentioned in Section 2.2.2. Furthermore, polysaccharides were also isolated by the combined use of graded alcohol precipitation and membrane separation as mentioned in Section 2.2.2. Finally, polysaccharides extracted from sweet tea leaves by MDAE (P-DM) were obtained.

Secondly, a four-factor Box-Behnken design (BBD) was applied to further evaluate the effects of extraction parameters on the extraction yield of P-DM. The independent variables include extraction time (X1, 8, 10, 12 min), extraction power (X2, 480, 560, 640 W), content of water in DES (X3, 10, 20, 30%), and liquid–solid ratio (X4, 20, 30, 40 mL/g). In this study, 29 experimental runs with 5 central points were performed by BBD as shown in Table 1, and each experiment in this study was tested in triplicate. Then, the experimental data of BBD were analyzed by the statistical function of the Design Expert software 8.0.6. The obtained data were fitted by the second-order polynomial model, as follows [21],

$$Y=\beta 0+\overset{3}{\underset{i=1}{\sum }}\beta \mathrm{iXi}+\overset{3}{\underset{i=1}{\sum }}2{\beta \mathrm{iiXi}}^{2}+\overset{2}{\underset{i=1}{\sum }}\overset{3}{\underset{j=i+1}{\sum }}\beta \mathrm{ijXiXj}$$

where Y is the predicted response; Xi and Xj are different variables; and β0, βi, βii, and βij are the regression coefficients for intercept, linearity, square, and interaction, respectively.

### 2.3. Determination of Physicochemical and Structural Properties of Polysaccharides Extracted from Sweet Tea Leaves

The contents of total polysaccharides, uronic acids, proteins, and conjugated polyphenols in P-W and P-DM were estimated by colorimetric methods, as previously reported [5]. The molecular weights (*M_w_*) and molecular mass distributions (*M_w_/M_n_*) of P-W and P-DM were measured by size exclusion chromatography coupled with a multi-angle laser light scattering detection (SEC-MALLS) as previously reported [27]. Indeed, a TSKgel GMPWXL column (300 mm × 7.8 mm, i.d.) was utilized for the separation of P-W and P-DM, and the mobile phase was 0.9% of NaCl solution. Besides, the constituent monosaccharides of P-W and P-DM were also detected by HPLC (Thermo Fisher Scientific, Waltham, MA, USA) as formerly reported [20]. A C_18_ column (150 mm × 4.6 mm, 5 μm, Thermo Fisher Scientific, Waltham, MA, USA) was applied for the separation of monosaccharides. The mobile phase was a mixture of 0.1 M phosphate buffer solution (pH = 6.7) and acetonitrile (83: 17, *v/v*) at a flow rate of 1.0 mL/min. The wavelength of DAD was set at 245 nm. A mixed standard solution, containing Man, Rha, GlcA, GalA, Glc, Gal, Xyl, and Ara, was analyzed as described above. In addition, the functional groups and degrees of esterification (DE) of P-W and P-DM were also analyzed by FT-IR spectra (Thermo Fisher Scientific, Waltham, MA, USA) as previously reported [20]. Moreover, the ^1^H and ^13^C NMR spectra of P-W and P-DM were also recorded by a Bruker Ascend 600 MHz spectrometer (Bruker, Rheinstetten, Germany) as previously reported so as to further characterize the chemical structure [20].

### 2.4. Evaluation of Biological Functions of Polysaccharides Extracted from Sweet Tea Leaves

#### 2.4.1. Determination of In Vitro Antioxidant Capacities

The in vitro assays for antioxidant capacities, including ABTS, DPPH, and NO radical scavenging capacities as well as ferric reducing antioxidant power (FRAP), were carried out for the evaluation of the in vitro antioxidant capacities of P-W and P-DM as previously reported [15,20]. Briefly, for the determination of ABTS radical scavenging ability, the ABST radical cation working solution (200 μL) was mixed with 20 μL of each sample in a 96-well microplate to react at 30 °C for 20 min; for the determination of DPPH radical scavenging ability, the DPPH working solution (200 μL) was mixed with 20 μL of each sample in a 96-well microplate to react at 37 °C for 30 min in the dark; for the determination of NO radical scavenging ability, each sample (450 μL) was mixed with 50 μL of sodium nitroprusside (10 mM) to react at 25 °C for 3 h, and then 250 μL of Griess reagent was added. For the determination of FRAP, 100 μL of each sample was mixed with 100 μL of potassium ferricyanide (1%, *w/w*) at 50 °C for 20 min, and then 100 μL of trichloroacetic acid (10%, *w/v*) was added and centrifugated. Finally, both distilled water (100 μL) and ferric chloride (20 μL) were added to the supernatant (100 μL). Additionally, P-W and P-DM were measured at five different concentrations with deionized water as the blank control. Besides, both *Vc* and BHT were used as positive controls in this study. The absorbances of the reaction mixtures for ABTS, DPPH, NO, and FRAP were detected at 734, 517, 540, and 700 nm, respectively.

#### 2.4.2. Determination of In Vitro Anti-Diabetic Effects

The in vitro anti-diabetic effects of P-W and P-DM were evaluated through the determination of α-glucosidase inhibitory activities as previously reported [15]. Briefly, P-W and P-DM were measured at five different concentrations with deionized water and acarbose as the blank control and the positive control, respectively. The absorbance of the reaction mixture was detected at 405 nm.

#### 2.4.3. Determination of In Vitro Antiglycation Effects

Antiglycation activities of P-W and P-DM were measured by using an in vitro BSA-glucose model as previously reported [28]. The fluorescence of advanced glycation end-products (AGEs) was detected at 370 nm for excitation and 440 nm for emission. The antiglycation activities of P-W and P-DM were also measured at five different concentrations with aminoguanidine (AG) as a positive control.

#### 2.4.4. Determination of In Vitro Prebiotic Potentials

An in vitro fermentation model was applied for the determination of prebiotic effects of P-W and P-DM as previously reported [29]. Briefly, P-W and P-DM were added into the carbohydrate-free MRS broth for the evaluation of their effects on the growth of four probiotic strains, including *Lactobacillus fermentum* (CGMCC 1.15608), *Lactobacillus plantarum* (CGMCC 1.12974), *Lactobacillus rhamnosus* (ATCC 53103), and *Bifidobacterium adolensentis* (ATCC 15703). Fructo-oligosaccharide (FOS, 1.0%, *w/v*) was applied as the positive control. Each sample (1.0%, *w/v*) was also added to the basal MRS. Then, each probiotic strain was inoculated into the culture medium and incubated at 37 °C for 48 h. Especially, *B. adolensentis* (ATCC 15703), *L. fermentum* (CGMCC 1.15608), and *L. plantarum* (CGMCC 1.12974) were cultivated under anaerobic conditions. Finally, the optical density was measured at 600 nm, and the total short-chain fatty acids (SCFAs) produced by each probiotic strain were detected by gas chromatography coupled with a flame ionization detector as previously reported [29].

#### 2.4.5. Determination of In Vitro Immunostimulatory Activities

The in vitro immunostimulatory activities of P-W and P-DM were determined by using a RAW 264.7 macrophage model, as previously reported [30]. Briefly, RAW 264.7 cells were cultured in the RPMI-1640 medium. Then, the effects of P-W and P-DM (5, 20, 80, and 320 μg/mL) on the cell proliferation of RAW 264.7 cells were measured by the MTT colorimetric method. Furthermore, the content of nitric oxide (NO) released from RAW 264.7 cells was measured by Griess reagent. In addition, the cytokines, including interleukin-6 (IL-6) and tumor necrosis factor-α (TNF-α), secreted from RAW 264.7 cells were determined by ELISA kits based on the manufacturer’s procedures (NeoBioscience, Shenzhen, China).

### 2.5. Statistical Analysis

All experiments were operated in triplicate, and data were expressed in means ± standard deviations. Statistical analysis was performed by ANOVA followed by Duncan’s test. Values of *p* < 0.05 were regarded as statistically significant.

## 3. Results and Discussion

### 3.1. Optimization of Microwave-Assisted Deep Eutectic Solvent Extraction of Polysaccharides from Sweet Tea Leaves

#### 3.1.1. Single Factor Experimental Results

**Figure 1:**
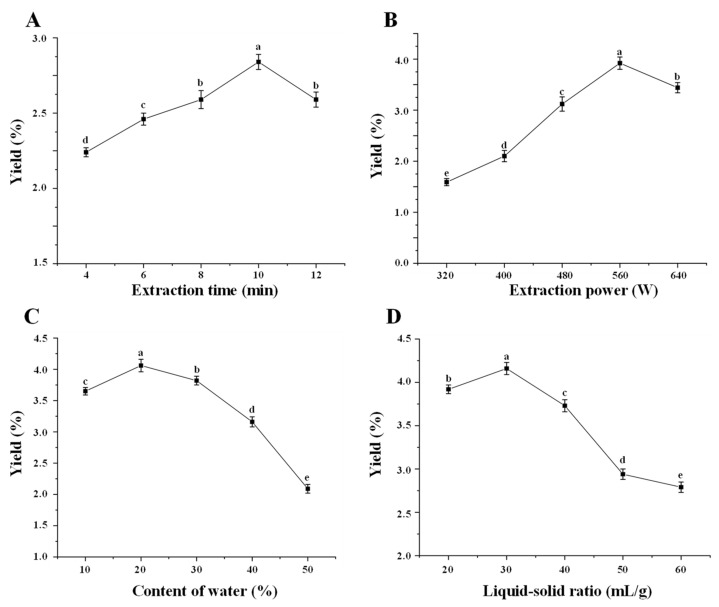
Effects of extraction time (**A**), extraction power (**B**), content of water (**C**), and liquid–solid ratio (**D**) on the yields of polysaccharides extracted from sweet tea leaves by microwave-assisted deep eutectic solvent extraction. Significant differences (*p* < 0.05) between samples are shown by data bearing different letters.

Usually, the extraction time, extraction power, and liquid–solid ratio are regarded as the main factors that affect the extraction efficiency of microwave-assisted extraction [31]. Therefore, the effects of extraction time, extraction power, content of water in DES, and liquid–solid ratio on the extraction yields of polysaccharides extracted from sweet tea leaves are shown in Figure 1. Figure 1A displays the effect of extraction time on the yield. The yields of P-DM improved with the increasing time ranging from 4 to 10 min and then declined with the increasing time ranging from 10 to 12 min. This phenomenon might be due to the degradation of P-DM under a long duration of microwave radiation [32]. As expected, the yields also improved with the extraction power increased from 320 to 560 W, while the yields of P-DM decreased with the increasing extraction power ranging from 560 to 640 W (Figure 1B). This phenomenon might result from the degradation of polysaccharides under excessively high microwave power, which could lead to the damage to chemical structures of natural polysaccharides [32,33]. Besides, different contents of the water in DES also obviously affected the extraction yields of P-DM. As shown in Figure 1C, the yields improved with the increase in the content of water ranging from 10% to 20% (*v/v*), while the yields decreased gradually with the increase of the content of water ranging from 20% to 50% (*v/v*). This phenomenon might be owing to the fact that the low content of water in DES makes it hard to penetrate the plant cell to extract polysaccharides because of its high viscosity, while the excess content of water in DES could decrease the interaction between DES and polysaccharides, resulting in a low extraction yield [21,24]. Furthermore, the effect of liquid–solid ratio on the yield of P-DM was also studied. As shown in Figure 1D, the yields of P-DM significantly improved with the increasing ratio ranging from 20 to 30 mL/g, while the yields obviously declined with the increase of ratio ranging from 30 to 60 mL/g. This result could be owing to the fact that a high liquid to solid ratio caused the notable decrease of microwave energy attached to the unit volume. Finally, the optimal extraction time, extraction power, content of water, and liquid to solid ratio were measured to be 10 min, 560 W, 20% (*v/v*), and 30 mL/g, respectively.

#### 3.1.2. Box-Behnken Experimental Results

According to the single factor experimental results, a Box-Behnken experimental design (BBD) was further utilized to optimize the conditions of MDAE. The BBD experimental data for the MDAE method are summarized in Table 1. Besides, a second-order polynomial equation was generated to express the mathematical model by using multiple regression analysis. The final equation was as follows,

$$Y=4.16+0.14X1+0.15X2+0.15X3-0.089X4+0.075X1X2-0.023X1X3+\;0.26X1X4-0.14X2X3-0.31X2X4+0.12X3X4-0.19X1^{2}-0.41X2^{2}-0.43X3^{2}-\;0.31X4^{2}$$

where Y is the predicted yield of P-DM (%, the dry weight of P-DM (g)/the dry weight of raw material (g)). X1, X2, X3 and X4 represent extraction time (min), extraction power (W), content of water in DES (%, *v/v*), and liquid to solid ratio (mL/g), respectively.

As shown in Table 2, the one-way ANOVA analysis was performed to confirm the significance of the extraction parameters on the yields and the predicted model. The fitted model was significant, which possessed a high *F*-value (36.44) and a very low *p*-value (*p* < 0.0001). This result indicated that the probability of error in this model was less than 0.01% [20]. Besides, the lack of fit was not significant, which possessed a low *F*-value (0.90) and a high *p*-value (0.5982). This result indicated that the fitted model equation could perfectly predict the yields of P-DM [19,20]. Besides, this fitted model was reproducible and reliable because of a low value of the coefficient variation (*CV*, 2.4%) and a high value of adequate precision (adeq. precision, 20.465) [20,21]. In addition, the predicted extraction yields of the fitted model were very close to the actually detected extraction yields according to the values of coefficient of determination (0.9733) and adjusted coefficient of determination (0.9466) [19,20]. Moreover, the linear coefficients (X1, X2, X3, and X4), interaction coefficients (X1X4, X2X3, X2X4, and X3X4), and quadratic term coefficients (X1^2^, X2^2^, X3^2^, and X4^2^) of this fitted model equation were significant with *P*-values lower than 0.05, while the interaction coefficients (X1X2 and X1X3) were not significant with *P*-values higher than 0.05 (Table 2). These results indicated that all selected extraction parameters could significantly affect the extraction yields of P-DM.

**Figure 2:**
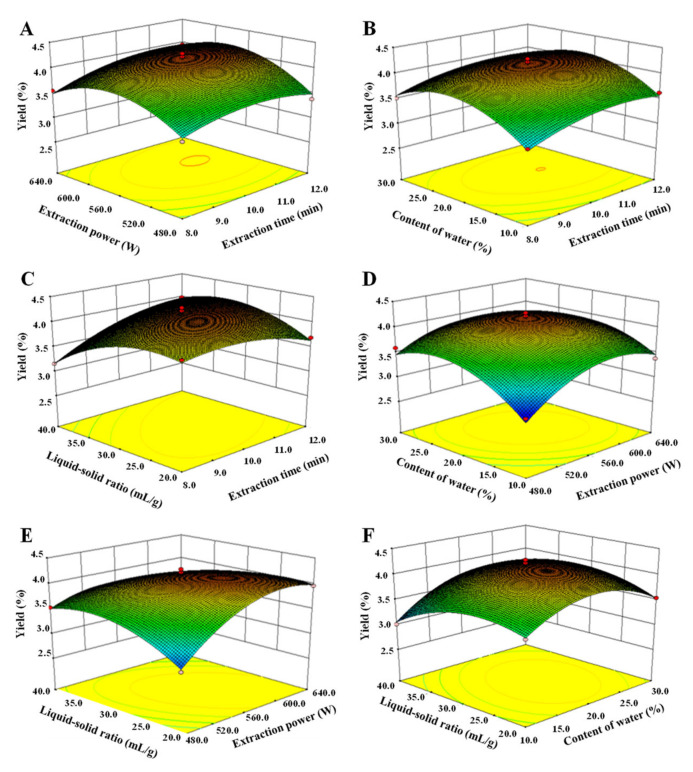
Three-dimensional response surface plots of microwave-assisted deep eutectic solvent extraction. **A**–**F**, interactions among extraction time, extraction power, content of water, and liquid–solid ratio, respectively.

**Figure 3:**
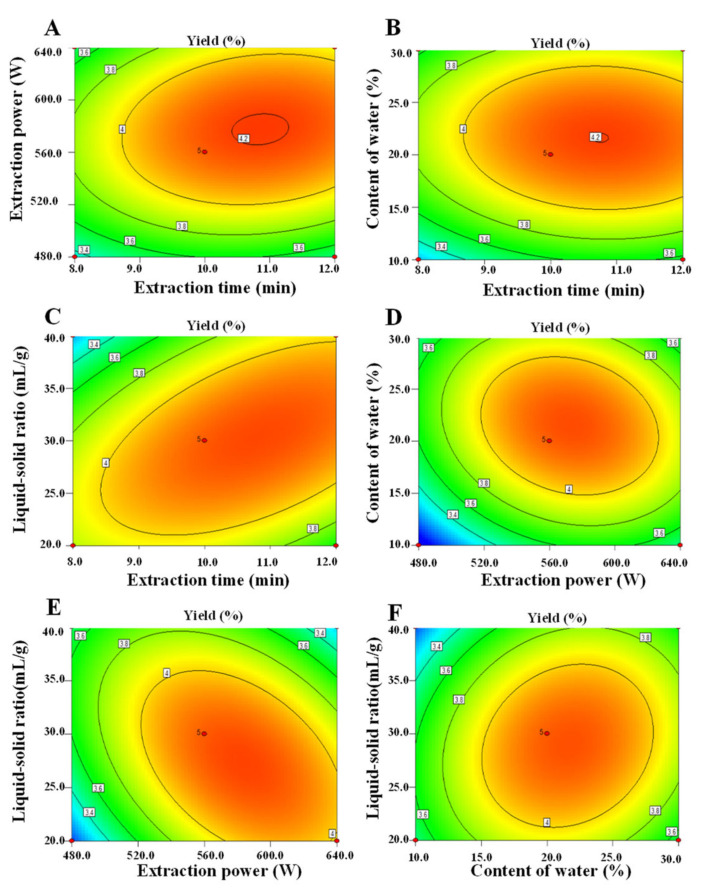
Two-dimensional contour plots of microwave-assisted deep eutectic solvent extraction. **A**–**F**, interactions among extraction time, extraction power, content of water, and liquid–solid ratio, respectively.

Moreover, the 3D response surface plots and the 2D contour plots of this fitted model equation are shown in Figure 2 and Figure 3. As shown in Figure 2, it could be predicted that the content of water in DES and extraction power possessed more positive influences on the yields of P-DM than those of extraction time and liquid to solid ratio [20]. Generally, the 2D contour plots can reflect the significance of mutual interaction effects among the variables [20,21], and the elliptical response contour plot is always related to significant interaction effects between the corresponding variables. As shown in Figure 3, it could be visually observed that interaction effects between the extraction time and the liquid–solid ratio, the extraction power and the content of water in DES, the extraction power and the liquid–solid ratio, and the content of water in DES and the liquid–solid ratio were significant, which could also be proved by the *p*-values of interaction coefficients (X1X4, X2X3, X2X4, and X3X4) as shown in Table 2. Furthermore, the predicted optimal extraction conditions are obtained by the analysis of the fitted model equation, which was as follows: extraction time of 10.74 min, extraction power of 577.75 W, water content in DES of 21.15% (*v/v*), and liquid–solid ratio of 29.23 mL/g. Nevertheless, considering the operability in the actual processing procedure, the verification experiment was carried out under the following conditions: extraction time of 11.0 min, extraction power of 576.0 W, water content in DES of 21.0%, and liquid–solid ratio of 29.0 mL/g. Under these conditions, the actual extraction yield of polysaccharides extracted from sweet tea by MDAE was 4.16% ± 0.09% (n = 3), which was in good agreement with the predicted extraction yield (4.22%). Compared with the HWE method in a previous study [5] and in the present study (Table 3), the optimized MDAE method possessed a higher extraction yield but a much shorter extraction time. The results indicated that the MDAE method could be applied as an efficient technique to extract polysaccharides from sweet tea leaves.

### 3.2. Physicochemical and Structural Characteristics of Polysaccharides Extracted from Sweet Tea Leaves by MDAE and HWE

#### 3.2.1. Chemical Compositions of Polysaccharides Extracted from Sweet Tea Leaves

The yields and compositions of P-DM and P-W are displayed in Table 3. Results displayed that the yield of P-DM under the optimal extraction conditions of MDAE was notably higher than that of P-W by HWE. However, both P-DM and P-W possessed high contents of total polysaccharides (85.48% and 81.30%, respectively) and low contents of total proteins (4.32% and 7.49%, respectively), suggesting that polysaccharides and minor polysaccharide-protein complexes might exist in P-DM and P-W [34,35]. Furthermore, total uronic acids in P-DM (43.43%) were significantly (*p* < 0.05) higher than that of P-W (38.19%). Obviously, the results indicated that the MDAE method could improve the extraction of acidic polysaccharides from sweet tea leaves. This phenomenon was similar to a previous study [20]. Additionally, minor conjugated polyphenols (43.26 mg GAE/g) were found in P-DM, which were also higher than that of P-W (20.36 mg GAE/g). This result might be owing to the favorable solubility of plant polyphenols in DES [36].

#### 3.2.2. Molecular Weights and Constituent Monosaccharides of Polysaccharides Extracted from Sweet Tea Leaves

**Figure 4:**
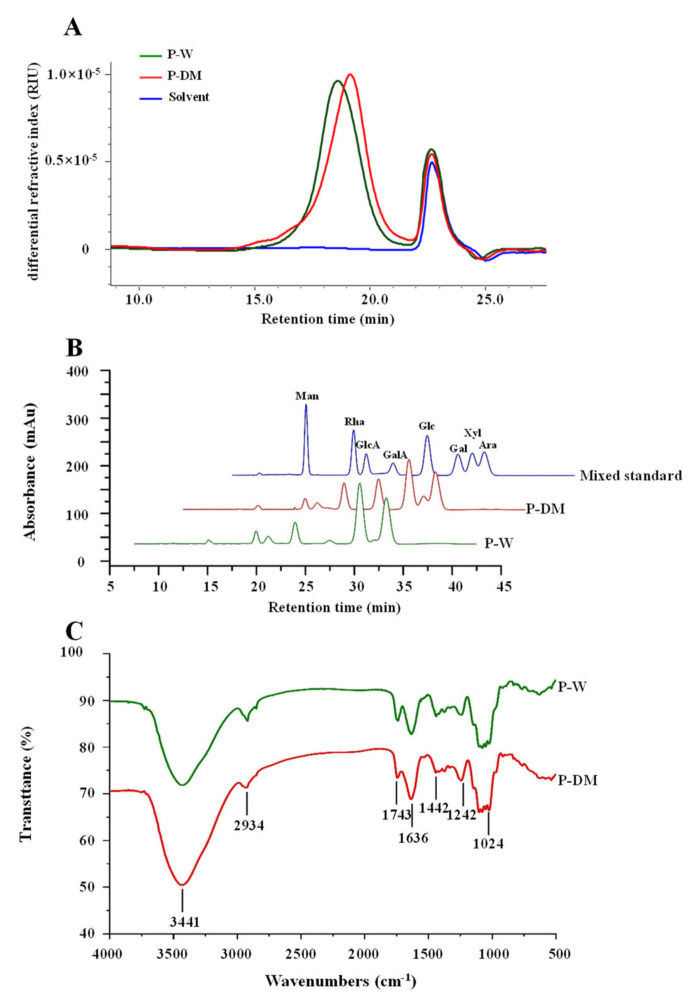
Size exclusion chromatograms (**A**), HPLC profiles of compositional monosaccharides (**B**), and FT-IR spectra (**C**) of polysaccharides extracted from sweet tea leaves. P-DM and P-W indicate polysaccharides extracted from sweet tea leaves by microwave-assisted deep eutectic solvent extraction and hot water extraction, respectively; Mixed standard, indicates GalA, Gal, Ara, Glc, Xyl, Rha, GlcA, and Man, which was analyzed by HPLC under the same conditions of samples.

Generally, it is considered that molecular weight and constituent monosaccharides make a profound and significant influence on the bioactivities of natural polysaccharides [27,30]. Consequently, the molecular weights and constituent monosaccharides of P-DM and P-W were investigated and compared. Figure 4A shows the HPSEC-RID chromatograms of P-W and P-DM. Results showed that both P-W and P-DM possessed only one symmetric polysaccharide fraction, indicating that the combined use of graded alcohol precipitation and membrane separation could effectively promote the purity of polysaccharides extracted from plants [37]. As displayed in Table 3, the molecular weight of P-W (2.541 × 10^5^ Da) was much higher than that of P-DM (1.575 × 10^5^ Da). The relatively low molecular weight of P-DM might be owing to the synergistic effect of microwave degradation [33] and targeted extraction by DES [20]. Meanwhile, the polydispersities of P-W and P-DM were 1.393 and 1.479, respectively, also suggesting that the molecular weight of P-DM might be degraded during microwave exposure [33].

Furthermore, the HPLC profiles of monosaccharides released from P-DM and P-W are shown in Figure 4B. Results showed that type of constituent monosaccharides in P-DM was the same as in P-W, which contained GalA, Gal, Ara, Glc, Xyl, Rha, GlcA, and Man. This result was in accordance with a previous study [5]. However, as summarized in Table 3, the molar ratio of monosaccharides in P-DM was quite different from that of P-W. Results showed that the molar ratios of GalA, Gal, Ara, Glc, Xyl, Rha, GlcA, and Man in P-W and P-DM were determined to be 1.57:1.23:1.00:0.07:0.06:0.29:0.23:0.04 and 2.22:1.23:1.00:0.77:0.34:0.25:0.21:0.05, respectively. It could be concluded that the MDAE method obviously affected the molar ratios of constituent monosaccharides, but hardly affected their types [20]. Obviously, GalA, Gal, and Ara were found as the main monosaccharides in both P-DM and P-W, suggesting that a large amount of homogalacturonan (HG) and arabinogalactan (AG) or arabinogalactan-protein complex (AGP) might exist in polysaccharides extracted from sweet tea leaves, as well as minor rhamnogalacturonan I (RG-I) might also exist based on the monosaccharide composition [5,20,38]. Besides, glucan might also exist in P-DM according to its relatively high molar ratio of Glc [38].

#### 3.2.3. FT-IR and NMR Spectra of Polysaccharides Extracted from Sweet Tea Leaves

In order to well understand the detailed structures of P-DM and P-W, both FT-IR and NMR analyses were performed. As displayed in Figure 4C, P-DM and P-W had similar functional groups because of their similar FT-IR spectra. The typical signals of pectic–polysaccharides, including 3441 cm^−1^, 2934 cm^−1^, 1743 cm^−1^, 1636 cm^−1^, 1442 cm^−1^, 1242 cm^−1^, and 1024 cm^−1^, were measured in both P-DM and P-W. More specifically, the absorption bands at 3441 cm^−1^ and 2934 cm^−1^ were owing to the stretching vibrations of O-H and C-H groups, respectively [22]. The signals at 1743 cm^−1^ and 1636 cm^−1^ were caused by the stretching vibrations of the esterified carboxylic groups and the free carboxylic groups, respectively [30], indicating the presence of uronic acids in P-DM and P-W. Indeed, the signal at 1442 cm^−1^ also corresponded to the presence of uronic acids [20]. Besides, the absorption band at 1242 cm^−1^ was caused by the stretching vibration of C-O-C, and the absorption band at 1024 cm^−1^ was related to the presence of pyranose sugar. Moreover, the DE values of P-W and P-DM were determined to be 38.22% and 31.23% based on the intensities of signals at 1743 cm^−1^ and 1636 cm^−1^, respectively, which indicated that the DE value of polysaccharides extracted from sweet tea was obviously affected by MDAE [5].

**Figure 5:**
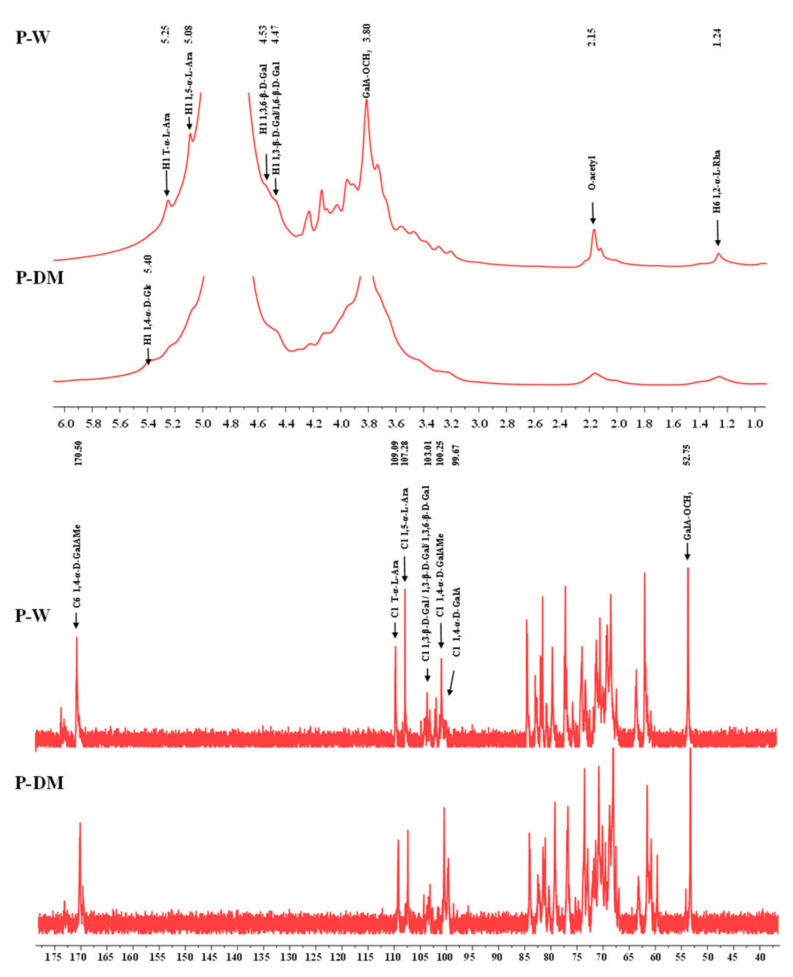
^1^H NMR and ^13^C NMR spectra of polysaccharides extracted from sweet tea leaves. P-DM and P-W indicate polysaccharides extracted from sweet tea leaves by microwave-assisted deep eutectic solvent extraction and hot water extraction, respectively.

As shown in Figure 5, both ^1^H and ^13^C NMR spectra of P-DM and P-W were similar, indicating that the primary structures of P-DM and P-W were similar. This result showed that the MDAE method did not change the primary structure of polysaccharides extracted from sweet tea [20]. More specifically, the signals at 5.26 ppm and 109.09 ppm suggested the presence of H-1 and C-1 of T-α-L-Ara*f* [39,40], respectively. The signals at 5.09 ppm and 107.28 ppm indicated the presence of H-1 and C-1 of 1,5-α-L-Ara*f* [34,39], respectively. Furthermore, the signals at 3.80 ppm and 52.75 ppm confirmed the presence of 6-O-methyl ester of GalA*p* (GalA-OCH_3_) [30,41,42], respectively. Indeed, the signals at 100.27 ppm and 170.50 ppm suggested the presence of C-1 and C-6 of 1,4-α-D-GalAMe*p* [41,42], respectively. The signals at 99.55 ppm and 173.61 ppm indicated the presence of C-1 and C-6 of 1,4-α-D-GalA*p* [20,30,41,42]. Notably, the relative intensity of the signal at 3.80 ppm was higher in P-W compared to P-DM, which indicated that the esterification degree of P-W was higher than that of P-DM. Additionally, the signals at 4.53 ppm and 104.27 ppm indicated the presence of H-1 and C-1 of 1,3,6-β-D-Gal*p* [34,40], the signals at 4.46 ppm and 103.10 ppm suggested the presence of H-1 and C-1 of 1,6-β-D-Gal*p*, and the signals at 4.46 ppm and 104.27 ppm suggested the presence of H-1 and C-1 of 1,3-β-D-Gal*p* [34,43]. The signals at 2.15 ppm and 1.24 ppm suggested the presence of O-acetyl groups and H-6 of 1,2-α-L-Rha*p*, respectively [30]. Moreover, the obvious signals at 5.40 ppm and 99.70 ppm were only found in the P-DM, which indicated that 1,4-α-D-Glc*p* might exist in P-DM [44]. This result was in accordance with the relatively high molar ratio of Glc detected in P-DM (Table 3). Collectively, these results indicated that the MDAE method did not alter the primary structure of polysaccharides extracted from sweet tea leaves, and a large amount of HG and AG might exist in P-DM and P-W.

### 3.3. In Vitro Antioxidant Capacities of Polysaccharides Extracted from Sweet Tea Leaves

**Figure 6:**
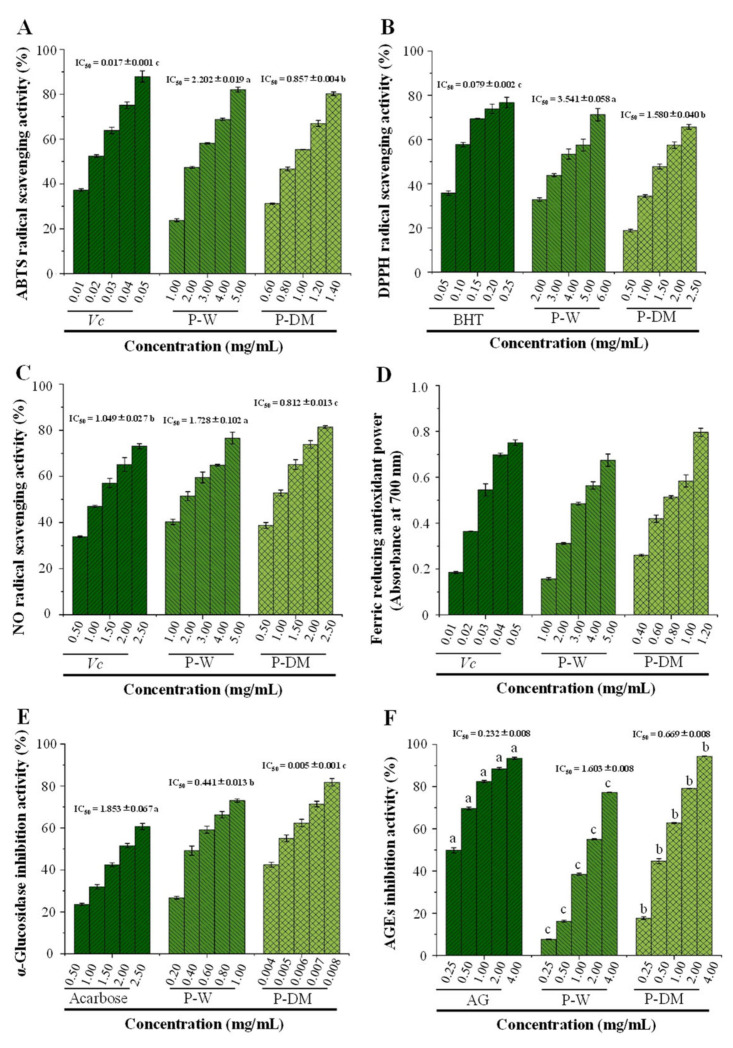
ABTS (**A**), DPPH (**B**) and NO (**C**)radical scavenging activities, reducing power (**D**), inhibitory activity against α-glucosidase (**E**), and antiglycation effect (**F**) of polysaccharides extracted from sweet tea leaves. P-DM and P-W indicate polysaccharides extracted from sweet tea leaves by microwave-assisted deep eutectic solvent extraction and hot water extraction, respectively; Significant differences (*p* < 0.05) among the positive control, P-DM, and P-W are shown by data bearing different letters (a–c).

In order to systematically evaluate the antioxidant capacities of P-DM and P-W, the assays for DPPH, ABTS, and NO radical scavenging abilities as well as the assay for FRAP were carried out in the present study. As displayed in Figure 6, compared with positive controls (*Vc* or BHT), both P-DM and P-W exhibited obvious antioxidant effects in a dose-dependent manner. More specifically, the IC_50_ values of ABTS, DPPH, and NO radical scavenging abilities of P-W and P-DM were determined to be 2.202 ± 0.019 mg/mL and 0.857 ± 0.004 mg/mL, 3.851 ± 0.046 mg/mL and 1.58 ± 0.04 mg/mL, and 1.728 mg/mL ± 0.102 and 0.812 ± 0.013 mg/mL, respectively, indicating that the antioxidant capacities of P-DM were about two times higher than P-W. Furthermore, as displayed in Figure 6D, the FRAP of P-W and P-DM (the absorbance at 700 nm) were measured to be 0.158 ± 0.005 and 0.584 ± 0.026 at the concentration of 1.0 mg/mL, further demonstrating that P-DM exerted significantly (*p* < 0.05) higher in vitro antioxidant activities than P-W.

Several studies have found that in vitro antioxidant capacities of polysaccharides extracted from plants are closely related to their physicochemical properties, like molecular weight, free uronic acids, conjugated polyphenols, and degree of esterification [5,15,20,28,30]. Usually, the lower molecular mass is always related to the stronger antioxidant capacity of acidic polysaccharides [30,45]. The higher contents of free uronic acids and conjugated polyphenols are also associated with higher antioxidant activity [20,28,46]. Therefore, in this study, P-DM extracted by MDAE exhibited notably higher in vitro antioxidant activities compared to P-W, which might be owing to its low molecular mass, high content of free uronic acids, and high content of conjugated polyphenols. Results indicated that the MDAE method was beneficial for the extraction of polysaccharides from sweet tea leaves with relatively high antioxidant capacity, and the P-DM could be explored as natural antioxidants for improving human health.

### 3.4. In Vitro Anti-Diabetic Effects of Polysaccharides Extracted from Sweet Tea Leaves

The inhibition of α-glucosidase activity can be a major strategy to counteract metabolic alterations associated with type 2 diabetes [30,47]. Sweet tea has been utilized as an herbal tea to prevent and manage diabetes in folk for a long history [1,2]. Indeed, its dihydrochalcones and polysaccharides have remarkable inhibitory effects on α-glucosidase in vitro [1,5]. Thus, the influence of the MDAE method on the in vitro α-glucosidase inhibitory activities of sweet tea polysaccharides was evaluated. As shown in Figure 6E, both P-W and P-DM exhibited notable inhibitory effects against α-glucosidase compared to acarbose. The IC_50_ values of P-W and P-DM inhibited α-glucosidase were detected to be 440.604 μg/mL and 4.629 μg/mL, respectively. Obviously, the inhibitory rate of P-DM on α-glucosidase was about 95 times that of P-W, and about 400 times that of acarbose, suggesting that P-DM has potential applications as a natural inhibitor against α-glucosidase for the prevention and management of type two diabetes.

According to previous studies, natural acidic-polysaccharides can inhibit the catalytic ability of α-glucosidase by binding with the enzyme to alter its structure [20,30]. In addition, natural polysaccharides with low molecular weights and high content of uronic acids can increase the binding capacity [20,48]. Besides, the polyphenols extracted from sweet tea also exhibited an obvious in vitro anti-diabetic effect through the inhibition of α-glucosidase [1,48]. Therefore, in the present study, the stronger α-glucosidase inhibitory effects of P-DM might be owing to its relatively lower molecular mass, higher content of uronic acids, and higher content of total polyphenols (Table 1).

### 3.5. In Vitro Antiglycation Effects of Polysaccharides Extracted from Sweet Tea Leaves

AGEs can be produced by interactions between free amino acids of proteins or lipids and reducing sugars [49]. AGEs can cause oxidative stress response and cell damage, leading to the occurrence and development of various diseases, such as diabetes and its complications [50]. The decrease in AGEs production may be beneficial for the prevention and alleviation of diabetes and its complications. In this study, the influence of the MDAE method on the in vitro antiglycation activity of sweet tea polysaccharides was further determined and compared. As shown in Figure 6F, both P-W and P-DM exhibited remarkable in vitro antiglycation activities compared to the positive control (AG). Indeed, the IC50 values of P-W and P-DM were detected to be 1.603 ± 0.008 and 0.669 ± 0.008 mg/mL, respectively. As expected, the antiglycation rate of P-DM was detected to be 94.44% at the concentration of 4.00 mg/mL, which was higher than that of P-W (77.27%). Previous studies have shown that the antiglycation activities of natural polysaccharides are closely related to their antioxidant activities, and the conjugated polyphenols can also contribute to their antiglycation effects [5,28].

### 3.6. In Vitro Prebiotic Effects of Polysaccharides Extracted from Sweet Tea Leaves

**Figure 7:**
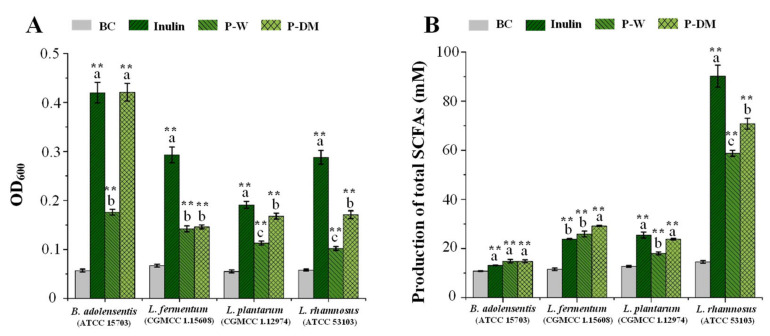
Effects of sweet tea polysaccharides on the growth (**A**) of four probiotic strains and related impacts on production of total short-chain fatty acids (**B**). P-DM and P-W indicate polysaccharides extracted from sweet tea leaves by microwave-assisted deep eutectic solvent extraction and hot water extraction, respectively; Significant differences (*p* < 0.05) among the positive control, P-DM, and P-W are shown by data bearing different letters (a–c). Significant differences in the growth of probiotic strains and production of total short-chain fatty acids in the positive control, P-DM, and P-W vs. blank control (BC) are shown by ** *p* < 0.01.

A large number of studies have revealed that non-starch polysaccharides isolated from edible and medicinal plants exert prebiotic potentials in vitro and in vivo, which can selectively modulate the gut microbiota to improve intestinal health [27,51,52]. Therefore, the effect of P-W and P-DM on the growth of several probiotics was investigated. As shown in Figure 7A, both P-W and P-DM could significantly promote the growth of *L. fermentum* (CGMCC 1.15608), *L. rhamnosus* (ATCC 53103), *L. plantarum* (CGMCC 1.12974), and *B. adolensentis* (ATCC 15703). More specifically, the prebiotic effect of P-DM on the growth of *B. adolensentis* was similar to that of the positive control (FOS), indicating that P-DM had good potential to be developed as natural prebiotics in the functional food industry. Besides, the prebiotic effects of P-DM on the growth of *B. adolensentis*, *L. plantarum*, and *L. rhamnosus* were notably higher than those of P-W, which might be closely related to its relatively low molecular weight [27]. Additionally, as displayed in Figure 7B, both P-W and P-DM could also promote the production of SCFAs from these probiotics, especially *L. rhamnosus* (ATCC 53103). Indeed, P-DM could induce *L. fermentum*, *L. plantarum*, and *L. rhamnosus* to generate higher contents of total SCFAs than that of P-W, which further indicated that P-DM could be developed as natural prebiotics for the improvement of intestinal health.

### 3.7. In Vitro Immunostimulatory Activities of Polysaccharides Extracted from Sweet Tea Leaves

**Figure 8:**
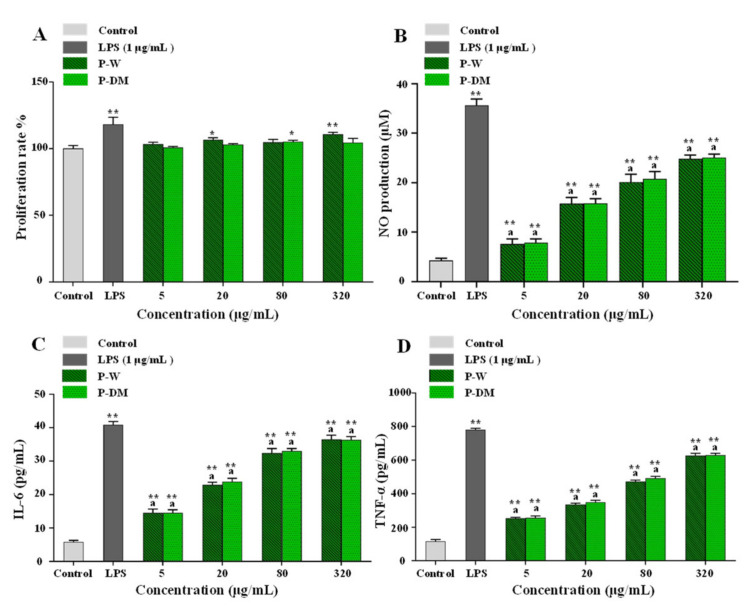
Effects of sweet tea polysaccharides on the proliferation (**A**), NO production (**B**), and cytokines (IL-6, **C**; TNF-α, **D**) released of RAW 264.7 cells. P-DM and P-W indicate polysaccharides extracted from sweet tea leaves by microwave-assisted deep eutectic solvent extraction and hot water extraction, respectively; Significant differences in cell proliferation and productions of NO, IL-6, and TNF-α in LPS, P-DM, and P-W vs. control are shown by * *p* < 0.05, ** *p* < 0.01. Significant differences (*p* < 0.05) in productions of NO, IL-6, and TNF-α between P-DM and P-W are shown by data bearing different letters.

As is known to all, immune cells play an important role in the immune system. Lots of studies have demonstrated that natural polysaccharides extracted from edible and medicinal plants can keep human health according to the regulation of the immune system [53]. Therefore, the immunostimulatory activities of P-W and P-DM on RAW 264.7 macrophages were investigated, including cell proliferation, NO production, and release of cytokines (IL-6 and TNF-α). As shown in Figure 8A, both P-DM and P-W showed no cytotoxic effects on RAW 264.7 cells at concentrations ranged from 5 to 320 μg/mL. Besides, as shown in Figure 8B–D, both P-DM and P-W notably promoted the production of NO, and the release of IL-6 and TNF-α from RAW 264.7 cells. At the concentration of 320 μg/mL, the productions of NO, IL-6, and TNF-α from RAW 264.7 cells activated by P-W and P-DM were 24.82 ± 0.80 and 25.07 ± 0.69 μM, 36.54 ± 1.26 and 36.38 ± 1.00 pg/mL, and 626.21 ± 14.12 and 628.88 ± 10.88 pg/mL, respectively. Results showed that the in vitro immunostimulatory effects of P-W and P-DM were similar.

Generally, the immunostimulatory effects of natural polysaccharides are closely related to the structural characters, such as molecular mass, the content of uronic acids, branched chain length, and esterified degree [54]. In fact, the immunostimulatory effects of acidic polysaccharides are positively associated with their esterified degree and content of uronic acids [20]. Besides, several studies have suggested that natural polysaccharides with low molecular weights often exert strong immunostimulatory effects [55,56]. Collectively, results showed that the immunostimulatory effects of P-W and P-DM were similar, which might be caused by the combined effect of several chemical properties, including molecular weights, contents of uronic acids, and esterified degree. There might be a balance among the contributions of the degree of esterification, the content of uronic acids, and the molecular weight to the immunostimulatory effect.

## 4. Conclusions

Polysaccharides are considered major bioactive components in sweet tea leaves. However, the knowledge about the chemical structures and biological functions of polysaccharides from sweet tea leaves is still unclear, which limits its application in the functional food industry. Therefore, in order to explore and promote the application of sweet tea polysaccharides in the functional food industry, the microwave-assisted deep eutectic solvent extraction of polysaccharides from sweet tea leaves was firstly optimized, and the structural properties and biological functions of sweet tea polysaccharides prepared by MDAE (P-DM) were investigated and compared with that of hot water extraction (P-W). The maximum extraction yield (4.16% ± 0.09%) of P-DM extracted by MDAE was obtained. Compare with hot water extraction, the optimized MDAE method possessed a higher extraction yield but a much shorter extraction time. Additionally, the MDAE method did not change the primary chemical structure of sweet tea polysaccharides. In fact, similar constituent monosaccharides were found in P-DM and P-W, and a larger amount of HG and AG might exist in both P-W and P-DM. Furthermore, P-DM exhibited higher in vitro antioxidant, anti-diabetic, antiglycation, and prebiotic effects than those of P-W, which might be closely associated with its lower molecular mass, higher content of free uronic acids, and higher content of conjugated polyphenols. Collectively, the findings indicate that the MDAE can be an efficient method for the preparation of polysaccharides with high bioactivities from sweet tea leaves, which are also helpful for good understanding of the potential structure–function relationship of sweet tea polysaccharide. Indeed, P-DM has good potential to be developed as functional food ingredients in the food industry, such as natural antioxidants, natural inhibitors against α-glucosidase, and natural prebiotics.

## Figures and Tables

**Table 1:** BBD with independent variables and observed values for the yields of polysaccharides extracted from sweet tea leaves by microwave-assisted deep eutectic solvent extraction.

Experiments	Levels of Extraction Parameters				Extraction Yields (%, *w/w*)
	X1 (min)	X2 (W)	X3 (%, *v/v*)	X4 (mL/g)	
1	0 (10)	0 (560)	−1 (10)	−1 (20)	3.45
2	1 (12)	−1 (480)	0 (20)	0 (30)	3.38
3	1 (12)	0 (560)	1 (30)	0 (30)	3.78
4	0 (10)	−1 (480)	1 (30)	0 (30)	3.59
5	1 (12)	0 (560)	0 (20)	1 (40)	3.98
6	0 (10)	−1 (480)	0 (20)	−1 (20)	3.02
7	0 (10)	−1 (480)	−1 (10)	0 (30)	2.96
8	0 (10)	0 (560)	0 (20)	0 (30)	4.08
9	1 (12)	0 (560)	0 (20)	−1 (20)	3.69
10	−1 (8)	0 (560)	−1 (10)	0 (30)	3.26
11	0 (10)	1 (640)	0 (20)	−1 (20)	3.96
12	1 (12)	0 (560)	−1 (10)	0 (30)	3.62
13	−1 (8)	0 (560)	0 (20)	1 (40)	3.16
14	−1 (8)	−1 (480)	0 (20)	0 (30)	3.28
15	−1 (8)	0 (560)	0 (20)	−1 (20)	3.91
16	0 (10)	0 (560)	0 (20)	0 (30)	4.16
17	0 (10)	0 (560)	−1 (10)	1 (40)	3.01
18	0 (10)	0 (560)	1 (30)	1 (40)	3.58
19	0 (10)	0 (560)	0 (20)	0 (30)	4.22
20	0 (10)	1 (640)	0 (20)	1 (40)	3.24
21	1 (12)	1 (640)	0 (20)	0 (30)	3.95
22	−1 (8)	0 (560)	1 (30)	0 (30)	3.51
23	0 (10)	1 (640)	−1 (10)	0 (30)	3.38
24	0 (10)	1 (640)	1 (30)	0 (30)	3.45
25	0 (10)	0 (560)	0 (20)	0 (30)	4.28
26	0 (10)	0 (560)	1 (30)	−1 (20)	3.54
27	0 (10)	−1 (480)	0 (20)	1 (40)	3.53
28	0 (10)	0 (560)	0 (20)	0 (30)	4.07
29	−1 (8)	1 (640)	0 (20)	0 (30)	3.55

X1: extraction time (min); X2: extraction power (W); X3: content of water (%, *v/v*); X4: liquid–solid ratio (mL/g).

**Table 2:** Analysis of the variance for the fitted second-order polynomial model for microwave-assisted deep eutectic solvent extraction.

	Sum of Squares	df	Mean Square	*F*-Value	*p*-Value
Model	3.84	14	0.27	36.44	<0.0001 **
X1	0.25	1	0.25	33.18	<0.0001 **
X2	0.26	1	0.26	34.73	<0.0001 **
X3	0.26	1	0.26	34.73	<0.0001 **
X4	0.095	1	0.095	12.69	0.0031 **
X1X2	0.023	1	0.023	2.99	0.1056
X1X3	2.025 × 10^−3^	1	2.025 × 10^−3^	0.27	0.6119
X1X4	0.27	1	0.27	35.97	<0.0001 **
X2X3	0.078	1	0.078	10.43	0.0061 **
X2X4	0.38	1	0.38	50.31	<0.0001 **
X3X4	0.058	1	0.058	7.66	0.0151 *
X1^2^	0.23	1	0.23	30.52	<0.0001 **
X2^2^	1.09	1	1.09	145.46	<0.0001 **
X3^2^	1.20	1	1.20	159.97	<0.0001 **
X4^2^	0.64	1	0.64	84.58	<0.0001 **
Residual	0.11	14	7.518 × 10^−3^		
Lack of fit	0.073	10	7.277 × 10^−3^	0.90	0.5982
Pure error	0.032	4	8.120 × 10^−3^		
Correlation total	3.94	28			

*R*^2^ = 0.9733, *R*^2^_adj_ = 0.9466, coefficient of variation (*CV*) = 2.4%, and adeq. precision =20.465; X1: extraction time (min); X2: extraction power (W); X3: content of water (%, *v/v*); X4: liquid–solid ratio (mL/g); * *p* < 0.05, ** *p* < 0.01.

**Table 3:** Comparison of chemical compositions, molecular weight (*M_w_*), polydispersity (*M_w_/M_n_*), and molar ratios of constituent monosaccharides of polysaccharides extracted by hot water extraction and microwave-assisted deep eutectic solvent extraction.

	P-W	P-DM
Yields and chemical compositions		
Extraction yields (%, *w/w*)	3.46 ± 0.11 ^b^	4.16 ± 0.09 ^a^
Total polysaccharides (%, *w/w*)	81.30 ± 1.40 ^a^	85.48 ± 0.36 ^b^
Total uronic acids (%, *w/w*)	38.19 ± 0.41 ^b^	43.43 ± 0.93 ^a^
Total proteins (%, *w/w*)	7.49 ± 0.10 ^a^	4.32 ± 0.05 ^b^
TPC (mg GAE/g)	20.36 ± 0.75 ^b^	43.26 ± 0.69 ^a^
Degree of esterification (%)	38.22 ± 1.32 ^a^	31.23.90 ± 0.59 ^b^
Molecular weight and its distribution		
*M_w_* × 10^5^ (Da, error)	2.541 ± 0.025 ^a^	1.575 ± 0.021 ^b^
*M_w_/M_n_*	1.393	1.479
Monosaccharides and molar ratios		
Galacturonic acid (GalA)	1.57	2.22
Galactose (Gal)	1.23	1.23
Arabinose (Ara)	1.00	1.00
Glucose (Glc)	0.07	0.77
Xylose (Xyl)	0.06	0.34
Rhamnose (Rha)	0.29	0.25
Glucuronic acid (GlcA)	0.23	0.21
Mannose (Man)	0.04	0.05

P-W and P-DM, polysaccharides extracted from sweet tea leaves by hot water extraction and microwave-assisted deep eutectic solvent extraction, respectively; TPC, total polyphenolic content; mg GAE/g, mg of gallic acid equivalent per gram of polysaccharides; Superscripts (a-b) differ significantly (*p* < 0.05) between P-W and P-DM.

## Data Availability

All of the Data is contained within the article.
